# Development and Characterization of a Novel *in vitro* Progression Model for UVB-Induced Skin Carcinogenesis

**DOI:** 10.1038/srep13894

**Published:** 2015-09-09

**Authors:** Nikhil Tyagi, Arun Bhardwaj, Sanjeev K. Srivastava, Sumit Arora, Saravanakumar Marimuthu, Sachin K. Deshmukh, Ajay P. Singh, James E. Carter, Seema Singh

**Affiliations:** 1 Department of Oncologic Sciences, Mitchell Cancer Institute, University of South Alabama, Mobile, Alabama 36604, USA; 2 Department of Biochemistry and Molecular Biology, College of Medicine, University of South Alabama, Mobile, Alabama 36688, USA; 3 Department of Pathology, College of Medicine, University of South Alabama, Mobile, Alabama 36688, USA

## Abstract

Epidemiological studies suggest ultraviolet B (UVB) component (290–320 nm) of sun light is the most prevalent etiologic factor for skin carcinogenesis- a disease accounting for more than two million new cases each year in the USA alone. Development of UVB-induced skin carcinoma is a multistep and complex process. The molecular events that occur during UVB-induced skin carcinogenesis are poorly understood largely due to the lack of an appropriate cellular model system. Therefore, to make a progress in this area, we have developed an *in vitro* model for UVB-induced skin cancer using immortalized human epidermal keratinocyte (HaCaT) cells through repetitive exposure to UVB radiation. We demonstrate that UVB-transformed HaCaT cells gain enhanced proliferation rate, apoptosis-resistance, and colony- and sphere-forming abilities in a progressive manner. Moreover, these cells exhibit increased aggressiveness with enhanced migration and invasive potential and mesenchymal phenotypes. Furthermore, these derived cells are able to form aggressive squamous cell carcinoma upon inoculation into the nude mice, while parental HaCaT cells remain non-tumorigenic. Together, these novel, UVB-transformed progression model cell lines can be very helpful in gaining valuable mechanistic insight into UVB-induced skin carcinogenesis, identification of novel molecular targets of diagnostic and therapeutic significance, and *in vitro* screening for novel preventive and therapeutic agents.

Skin cancer, comprising of melanoma, basal cell carcinoma (BCC) and squamous cell carcinoma (SCC), is the most common form of cancer worldwide^1^. Moreover, incidence of skin cancer has continued to rise at an alarming rate over the past decades despite advancement in our understanding of its etiological causes and prevention awareness^2^,^3^. Epidemiological data suggest that every third cancer diagnosis is for skin cancer and collectively more people are diagnosed with skin malignancy than the combined incidence of all other cancer types^1^,^4^. According to the statistics of Skin Cancer Foundation, ∼3.5 million people were diagnosed with skin cancer in the United States alone^5^,^6^ and one out of five Americans develops skin cancer in his/her lifetime^5^,^6^. This high statistics poses significant economic burden, besides its morbidity and mortality to the cancer patients^7^. Clearly, we need a better understanding of the molecular causes and mechanisms involved in skin carcinogenesis in order to develop more effective prevention and therapeutic strategies.

It is well established that solar ultraviolet (UV) radiation is the main etiological factor in human skin carcinogenesis accounting for about 90% of non-melanoma skin cancer cases^8^,^9^. Solar UV radiation is classified into three categories; i) UVA (315–400 nm), ii) UVB (280–315 nm), and iii) UVC (100–280 nm). UVC is not reported to have a role in cancer incidence because of its complete absorption by the ozone layer. UVA, on the other hand, constitutes ∼95% of solar UV radiation, but considered far less carcinogenic based on its low DNA damaging capability^10^. UVB, although constituting only ∼5% of solar UV radiation, is ∼10,000 times more carcinogenic than UVA and thus, considered as the major cause of human skin carcinogenesis^11^. UVB is a complete environmental carcinogen capable of initiating, promoting and facilitating progression of skin cancer. It induces DNA damage by forming cyclobutane pyrimidine dimers (CPDs) and (6–4) pyrimidine-pyrimidine photoproducts, which largely lead to initiation of skin carcinogenesis^12^,^13^. It has also been shown that UVB-induced photolesions are major contributors of p53 mutations (50–90%) in human SCC^14^. However, it is yet unclear, how these genetic aberrations transform the keratinocytes into malignant ones and what progressive changes occur in the biology of skin cells.

In the present study, we have developed an *in vitro* cell line model of UVB-transformed immortalized human epidermal keratinocytes (HaCaT) cells. HaCaT sublines were developed by intermittent exposure to UVB radiation over several weeks that exhibit phenotypic differences consistent with oncogenic transformation. These HaCaT sublines can provide valuable *in vitro* model to gain insight into involved genetic and epigenetic aberrations and involved signaling pathways. This model may be useful in the testing of novel preventive and therapeutic strategies against skin cancer.

## Results

### HaCaT sublines developed by repetitive UVB irradiation exhibit altered morphology and enhanced growth characteristics

**Figure 1:**
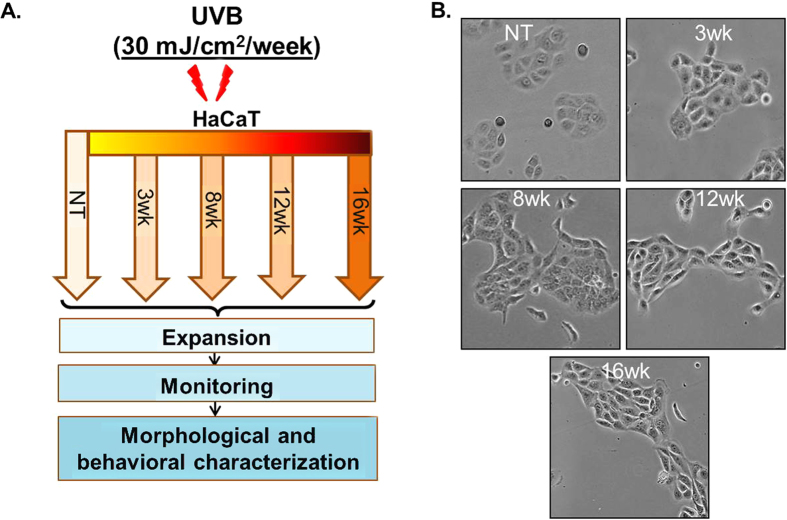
Schematic diagram showing the strategy for developing UVB-transformed HaCaT cell line model for skin cancer and their morphological characteristics. (**A**) HaCaT cells were continuously irradiated with UVB at dose of 30 mJ/cm^2^ once a week for 3, 8, 12 and 16 weeks and designated as 3wk, 8wk, 12wk and 16wk sublines, respectively. Non-UVB irradiated HaCaT (HaCaT-NT) cells were used as control. (**B**) Morphology of control and UVB-transformed HaCaT sublines was observed under phase-contrast microscope. Representative micrographs are from one of the random fields of view (magnification 200 X).

**Figure 2:**
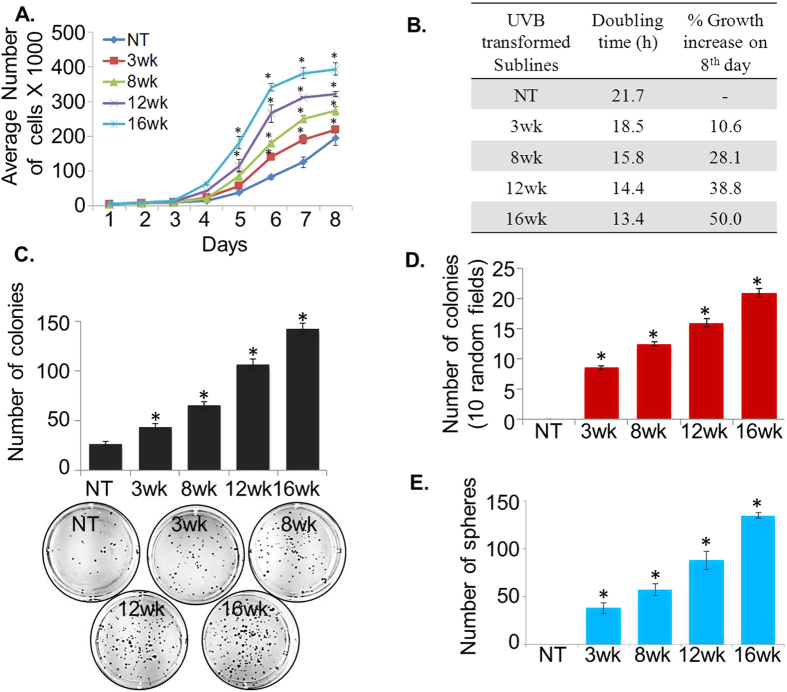
Growth characteristics of UVB-transformed HaCaT sublines. (**A**) Cells (1 × 10^4^ cell/well) were seeded in 6-well plate and growth was monitored for 8 days by counting viable cells. Growth curve represents data from triplicate experiments (mean ± SEM; n = 3, *p < 0.05). (**B**) The population doubling time and percent growth increase on 8^th^ day was calculated as described in materials and methods section. (**C**) Cells (1000 cells/well) from each subline were plated in 6-well plate and allowed to form colonies. After 2 weeks, colonies were stained with crystal violet, visualized, photographed and counted using imaging system. (**D**) Control and UVB-transformed HaCaT sublines were seeded at a density of 2.5 × 10^4^ cells/ml in a 0.4% soft agar over a 0.8% agar bottom layer. After 3 weeks, colonies were visualized and counted using Nikon eclipse microscope. Bars represent mean ± SEM; n = 3, *p < 0.05. (**E**) Single-cell suspensions of HaCaT sublines (1 × 10^3^ cells/well) were seeded in ultra-low attachment 6-well plate containing sphere formation medium (1:1, DMEM/F12) supplemented with B27, bFGF (10 ng/mL) and EGF (10 ng/mL). After 2 weeks, spheres were photographed and counted using Nikon Eclipse microscope.

To develop an *in vitro* model of UVB-induced skin carcinogenesis, we irradiated HaCaT cells to sub-erythemal dose of UVB radiation (30 mJ/cm^2^) once a week for up to 16 weeks. Following 3, 8, 12 and 16-weeks of UVB irradiation, HaCaT cells were expanded and cultured to attain stable phenotypes (Fig. 1A). Subsequently, we studied the morphology of all the UVB-irradiated and non-irradiated (parental) cells by phase-contrast microscopy. We observed striking differences in morphology of HaCaT sublines derived after repetitive UVB-irradiation. HaCaT cells that were irradiated for a longer repetitive exposure acquired more spindle or elongated shape as compared to parental cells, which was more uniform, smaller in size and rounder in shape (Fig. 1B). Further, monitoring of these sublines in subsequent passages demonstrated no reversal of these morphological characteristics. Next, we examined the growth, clonogenicity and sphere formation ability of these sublines. We observed that the growth rate of UVB-irradiated HaCaT (3wk, 8wk, 12wk and 16wk) sublines was significantly higher as compared to the parental HaCaT cells (Fig. 2A). Total number of cells on the 8^th^ day of culture indicated 10.6% (3wk), 28.1% (8wk), 38.8% (12wk) and 50.0% (16wk) increase in the growth of UVB-irradiated sublines as compared to the parental cells (Fig. 2A,B). Furthermore, cell population doubling time (dt) estimated during the logarithmic growth phase was also progressively decreased in derived cell lines subjected to greater repetitive exposure to UVB radiation (Fig. 2B). Further, in our plating efficiency assay, we observed that UVB-irradiated HaCaT sublines (3wk, 8wk, 12wk and 16wk) exhibited significantly higher (1.6, 2.5, 4.0, and 5.5 folds, respectively) plating efficiency as compared to the parental HaCaT cells (Fig. 2C). More importantly, while parental HaCaT cells did not form any colony in anchorage-independent (soft agar) clonogenicity assay, all UVB-irradiated HaCaT sublines exhibited clonogenic ability (Fig. 2D). The number of colonies formed by HaCaT sublines was directly correlated with the UVB-irradiation treatment length. Moreover, we also examined the sphere formation ability of these sublines by growing in ultra-low attachment plates containing sphere formation medium. Parental HaCaT cells did not form any sphere but in contrast UVB irradiated HaCaT cells formed spheres. Importantly, the sphere formation ability of HaCaT sublines increased with time of UVB irradiation (Fig. 2E). Taken together, these findings clearly suggest that UVB-transformed cells have gained increased growth characteristics, clonogenic potential and sphere-formation ability as compared to parental HaCaT cells.

### Increased growth of UVB-transformed HaCaT sublines is associated with enhanced proliferation and apoptosis-resistance

**Figure 3:**
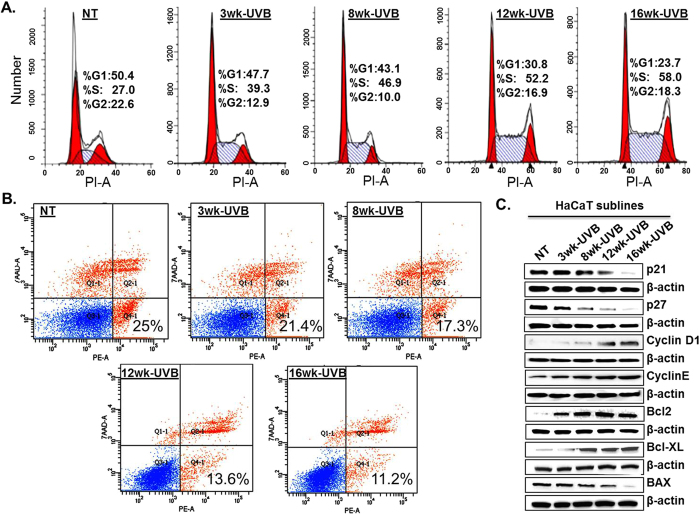
UVB-transformed HaCaT sublines exhibit enhanced proliferation and apoptosis resistance. (**A**) Control and UVB-transformed HaCaT cells (5 × 10^5^/well) were synchronized by growing in serum deprived medium for 72 h. Subsequently, cells were grown in fresh culture medium for 24 h. Thereafter, the cells were fixed and stained with Propidium iodide (PI) and distribution of cells in different phases of cell cycle was analyzed using flow cytometry. (**B**) Cells (5 × 10^5^/well) were seeded in 6-well plate. After 24 h cells were replenished with fresh medium and further allowed to grow for 96 h. After that cells were harvested and stained using PE Annexin V apoptosis detection kit. Percentage of apoptotic cells were analyzed by flow cytometry after staining. (**C**) Total protein from control and UVB-transformed HaCaT cells was prepared and expression of various proteins associated with cell cycle and apoptosis was examined by immunoblot analysis. β-actin was used as an internal control.

Having observed higher growth and clonogenic potential of UVB-transformed HaCaT sublines, we next examined their cell cycle distribution and apoptotic-index. We observe that all UVB-transformed HaCaT sublines have significantly higher cell cycle progression as compared to the parent cell line as is evident from greater distribution of cells in the S phase of cell cycle (Fig. 3A). Conversely, data from apoptosis assay demonstrate that UVB-transformed HaCaT cells are relatively more resistant to apoptosis as compared to the parent HaCaT cells (Fig. 3B). Notably, increase in S-phase distribution of the derived sublines positively correlated with their UVB exposure frequency (2.15 fold-16wk; 1.93 fold-12wk; 1.74 fold-8wk and 1.45 fold-3wk) (Fig. 3A), while an inverse association was observed for the apoptotic index (2.23 fold-16wk; 1.83 folds-12wk; 1.44 fold-8wk and 1.2 fold-3wk) (Fig. 3B).

A number of proteins are involved in the regulation of cell cycle and apoptosis at the molecular level^15^,^16^. Therefore, we analyzed the expression profile of these proteins in HaCaT and its derived sublines by immunoblot assay. The data show a progressive decrease in the expression of cyclin dependent kinase inhibitor proteins (p21 and p27) and pro-apoptotic protein i.e. Bax in UVB-transformed HaCaT sublines, whereas the expression of cyclins (Cyclin D1 and Cyclin E) and anti-apoptotic proteins (Bcl2 and Bcl-XL) was increased as compared to the parental HaCaT cells (Fig. 3C). Together, these findings suggest that UVB-transformed HaCaT cell lines exhibit enhanced proliferation rate and more resistance to apoptosis as compared to the parental cells.

### HaCaT sublines derived upon UVB-irradiation exhibit greater motility, invasiveness and epithelial-to-mesenchymal transition (EMT)

**Figure 4:**
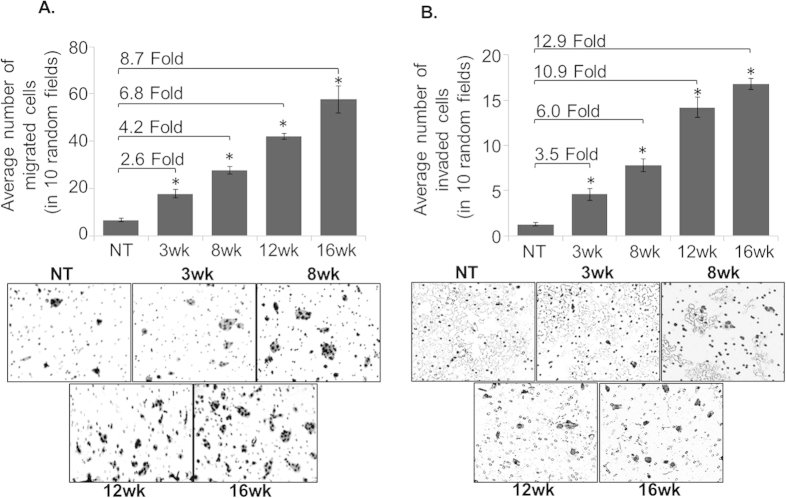
UVB-transformed HaCaT sublines depict increased motility and invasiveness. (**A**) Control and UVB-transformed HaCaT cells (2 × 10^5^/well) were seeded in the top chamber of non-coated polyethylene teraphthalate membrane and incubated for 16 h. Media containing 10% FBS was used as a chemo attractant. (**B**) Cells (5 × 10^4^/well) were plated in the top chamber of the trans-well with a Matrigel-coated polycarbonate membrane and incubated for 16 h. Media containing 10% FBS was used as a chemo attractant. Migrated/invaded cells were fixed, stained and counted in 10 random view fields. Bars represent the mean ± SEM; n = 3; *p < 0.05, magnification 100 X.

**Figure 5:**
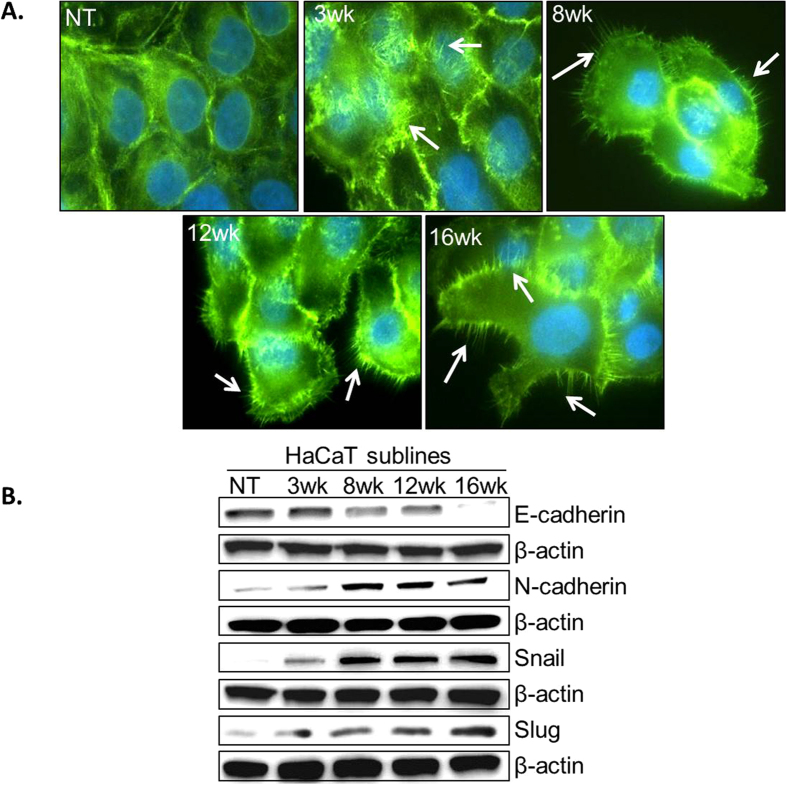
UVB-transformed HaCaT sublines exhibited mesenchymal characteristics. (**A**) Cells were grown overnight in Fluorodish, fixed and stained with FITC-conjugated phalloidin. After washing cells were mounted using DAPI containing Vectashield mounting medium. Cells were then analyzed and photographed (magnification 400 X) using fluorescent microscope . UVB-transformed cells exhibit several filopodia-like projections (white arrows) as compared to control HaCaT cells. (**B**) Expression profiles of various epithelial (E-cadherin) and mesenchymal (N-cadherin, Slug and Snail) markers were examined by immunoblot analyses. β-actin was used as an internal control.

In next set of experiments, we determined the motility and invasive potential of parental and UVB-transformed HaCaT sublines. Our data demonstrate that HaCaT sublines (3wk, 8wk, 12wk and 16wk) are more motile (2.6, 4.2, 6.8 and 8.7 folds, respectively) and invasive (3.5, 6.0, 10.9, and 12.9 folds, respectively) as compared to the parental HaCaT cells (Fig. 4A,B). Since acquisition of motile behavior and invasiveness is associated with transition to mesenchymal phenotype^17^,^18^, we next analyzed the cytoarchitecture of HaCaT and derived sublines by staining them with FITC-conjugated phalloidin. Microscopic examination demonstrated actin reorganization in UVB-transformed HaCaT sublines characterized by the presence of distinctive filopodia like structures (Fig. 5A). To further confirm the EMT of HaCaT sublines, we examined the expression of EMT-associated marker proteins. We observed a greater expression of mesenchymal markers (N-cadherin, slug and snail) and reduced expression of epithelial marker (E-cadherin) in HaCaT sublines as compared to parental cells (Fig. 5B). Together, these findings suggest that UVB-transformed sublines undergo EMT, and attain a more motile and invasive phenotype.

### UVB- irradiated HaCaT cells exhibit tumorigenic potential in mice

**Figure 6:**
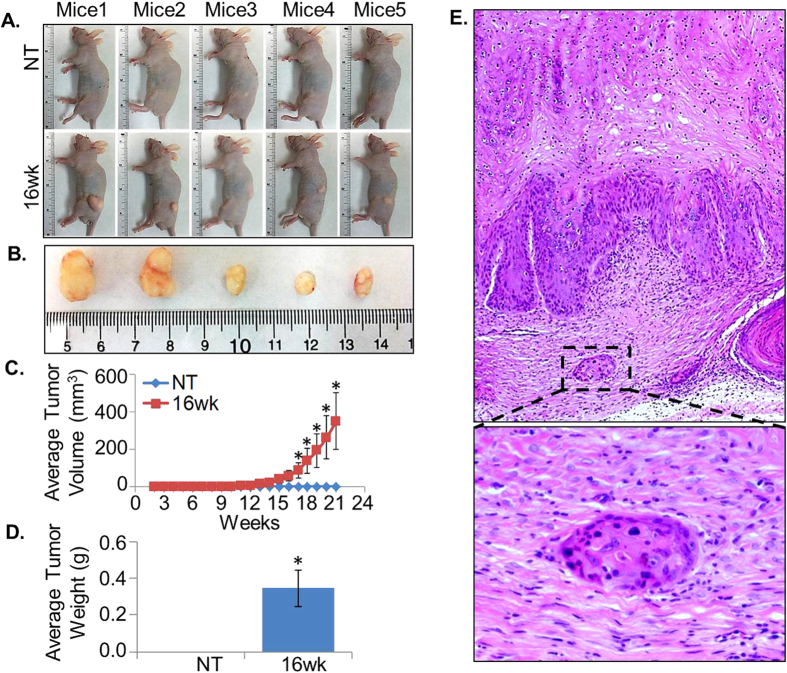
Tumorigenic competence of control and UVB-transformed cells in nude mice. HaCaT control (NT) and UVB-transformed (16wk) cells (1 × 10^6^) were injected into the left flank region of mice (n = 5, each group) and tumor growth was monitored for 21 weeks. (**A)** Pictures of mice of control (NT) and 16wk group (carrying tumors) after 21 weeks of implantation. (**B**) Excised tumors from 16wk cells after 21 weeks. (**C**) Tumor diameters were measured and tumor volume was calculated as described in Materials and Methods section. (**D**) Weight (mean ± SEM; n = 3, *p < 0.05) of the excised tumors. (**E**) H&E staining was performed on paraffin embedded tumor sections from 16 wk group mice, examined under microscope and photographed (magnification 200 X).

Next, we conducted *in vivo* study to examine if chronic UVB irradiation of HaCaT cells led to their oncogenic transformation. For this, we injected 16wk-HaCaT subline and parental HaCaT cell line subcutaneously into the immunocompromised nude mice and monitored the growth for 21 weeks. In accordance with the previously published studies^19^,^20^,^21^, we did not observe any tumor formation in mice injected with parental HaCaT cells even after 21 weeks, while there was 100% incidence of tumor formation in mice injected with 16wk-HaCaT subline (Fig. 6A,B). Tumor mass was visually evident at 10 week post-injection, which continue to increase until the experiment was ended (Fig. 6C). Average volume and weight of the developed tumors were 468.5 mm^3^ (range from 180 to 1152 mm^3^) and 0.346 g (range from 0.2 to 0.7 g), respectively (Fig. 6C,D). Histological examination of tumor by H&E staining revealed the feature of well differentiated squamous cell carcinoma with multilayered, hyper proliferative, stratified epithelium exhibiting prominent parakeratosis (Fig. 6E). Interestingly, in some sections, we also observed the presence of tumor cell nest into the basal connective tissue (Fig. 6E) suggesting the invasive nature of developed tumors. Together, these findings demonstrate that frequent exposure of UVB radiation causes oncogenic transformation of HaCaT cells, and derived subline forms well differentiated invasive squamous cell carcinoma.

## Discussion

Epidermal keratinocytes are the most predominant (∼95%) cell type present in the outermost layer of skin and therefore are the prime target of UVB radiations^22^,^23^. The present study developed and characterized a unique and stable *in vitro* progression model of UVB-induced skin carcinogenesis by using HaCaT cells. The most common scenario of UV exposure that could be associated with skin carcinogenesis in human is the chronic/repetitive exposure of keratinocytes to UV from sunlight during recreational sun bathing activities or from tanning beds^24^,^25^. According to published studies, around 40 mJ/cm^2^ of UVB is a minimal erythemal dose^26^,^27^, which causes DNA damage and significant apoptosis in exposed keratinoytes^28^. Therefore, we chose a sub-erythemal dose (30 mJ/cm^2^) of UVB and allowed repetitive exposure of keratinocytes to reflect a practical scenario of repetitive DNA damage and repair. Consequently, our treatment led progressive visible changes in cellular morphology as would be expected during oncogenic transformation^29^. Besides alteration in morphology, UVB-exposed sublines also exhibited enhanced growth, clonogenic potential and sphere-forming capacity as compared to the control HaCaT cells. Published study suggest that UVB at low doses (2.5 to 10 mJ/cm^2^) induces HaCaT cell proliferation without noticeable cell death^30^, whereas a single exposure at higher does (>20 mJ/cm^2^) inhibits proliferation and survival^23^,^31^. However, we did not see any growth stimulation in our study (data not shown) even at low UVB doses, but observed noticeable death of HaCaT cells at higher doses (20–40 mJ/cm^2^). Therefore, enhanced growth of remaining HaCaT cells upon repetitive UVB-exposure in our study is likely due to the accumulation of deleterious mutations and/or activation of tumor-promoting signaling pathways in these cells.

We also observed increased cell-cycle progression and apoptosis resistance, the key characteristics of a tumor cell, in UVB-transformed HaCaT sublines. Our results are consistent with several previously published reports that also highlight the association of increased cell-cycle progression and apoptotic resistance with the enhanced growth potential of a cell^30^,^32^,^33^. At molecular level cell-cycle process is tightly regulated by specific proteins cyclins, cyclins-dependent kinases (CDKs) and inhibitors of CDKs^15^,^16^. Similarly, a fine balance of pro- and anti- apoptotic proteins regulates the cellular apoptosis^34^. Higher expression of cyclins (Cyclin D1 and E) and anti-apoptotic proteins (Bcl2 and Bcl-XL) and reduced expression of CDK inhibitors (p21 and p27) and pro-apoptotic proteins (Bax) in UVB-transformed cells may thus, provide the molecular basis for the enhanced proliferation and survival in these cells. In corroboration to these observations, Han and He (2009) have also demonstrated the increased G1-S phase cell-cycle progression in human keratinocytes upon UVB exposure that correlated with the increased expression of cyclin D1^30^. A study performed by Liang *et al.* (2000) provides direct support to our findings^35^, suggesting that increased expression of cyclin D1 is an early event in skin cancer, and its overexpression was further suggested to be associated with sun exposure. Moreover, altered expression of Bcl2 family member proteins including Bcl2 and Bax in skin tumors as compared to case-matched nonneoplastic skin samples has been observed, clearly suggesting their critical roles in skin carcinogenesis^36^.

Furthermore, the mesenchymal phenotype is directly associated with the aggressiveness of tumor cells and transition of a cell from epithelial to mesenchymal stage is considered as an important phenomena of tumor progression^17^,^37^. In agreement to this, we also observed that UVB-transformed HaCaT cells exhibiting aggressive phenotypes also had mesenchymal characteristics including high expression of N-cadherin, Slug and Snail as compared to their normal counterparts. It has been reported that UVB exposure results in loss of E-cadherin and compromised E-cadherin-beta-catenin signaling in HaCaT cells^38^. Further, role of Snail and Slug, the transcriptional repressors of E-cadherin, in promoting EMT has been well documented in a number of tumors including skin cancer^39^,^40^. Choudhary *et al.* (2013) investigated the effect of UVB-irradiation on EMT in UVB-induced tumors in SKH-1 hairless mice and found the elevated level of mesenchymal markers like N-cadherin, snail, slug and twist while reduced expression of epithelial marker E-cadherin in UVB-induced tumors^41^. Together, these studies clearly support our findings that UVB causes EMT and thus enhances aggressive phenotypes in HaCaT cells.

Our study also provided a convincing evidence of malignant transformation of HaCaT cells that were chronically exposed to UVB radiation. All the mice injected with UVB irradiated HaCaT subline developed tumors, whereas no tumor formation was observed in any of the mice implanted with control HaCaT cells. This is consistent with published data demonstrating non-tumorigenic nature of HaCaT cells^20^,^26^. On the other hand, studies have shown that long-term thermal stress or activation of stromal cells could potentially induce tumorigenic conversion of HaCaT cells^20^,^42^. The tumors developed by UVB-transformed HaCaT cells in our study were histologically well differentiated SCCs and aggressively invaded into the surrounding tissue. Similarly, earlier studies also showed tumorigenic conversion of HaCaT cells by chronic UVA exposure or deregulated Nf-κB signaling form well differentiated SCC and invasive tumors in nude mice^29^,^43^. However, only benign tumor formation has been reported in elevated temperature transformed HaCaT cells without any signs of local invasion that suggests the more aggressive nature of UVB transformed HaCaT cells^20^.

In conclusion, we have developed a novel *in vitro* model system for the UVB-induced skin carcinogenesis. Our data provides evidence that repetitive exposure of UVB alone at sub-erythemal doses can cause malignant transformation of human epidermal keratinocytes. This novel *in vitro* cell line model mimicking *in vivo* tumor promotion can be useful in dissecting progressive changes in genes and molecular signaling pathways responsible for initiation; progression and development of UVB-induced skin carcinogenesis. The model can also facilitate identification of novel molecular diagnostic/therapeutic/preventive targets, and be useful for the *in vitro* screening for the agents against UVB-induced skin malignancies.

## Materials and Methods

### Cell culture

Immortalized human epidermal keratinocyte (HaCaT) obtained from German Cancer Research Center (Heidelberg, Germany) were maintained as monolayer culture in a humidified atmosphere with 5% CO_2_ at 37 °C in Dulbecco Modified Eagle Medium (DMEM; Invitrogen, Carlsbad, CA) supplemented with 10% (v/v) fetal bovine serum (FBS; Atlanta Biologicals, Lawrenceville, GA), penicillin (100 units/mL) and streptomycin (100 μg/mL) (Invitrogen). For sphere formation assay, cells were grown in DMEM/F12 medium (1:1, Invitrogen) supplemented with B-27, bFGF and EGF (Life technology™, Carlsbad, CA). Cells were routinely tested and determined to be free from mycoplasma contamination using MycoSensorPCR assay kit (Stratagene, La Jolla, CA) as per manufacturer’s protocol. Short tandem repeats (STR) genotyping was used as a way to authenticate the cell lines. The STR profiling was matched to the Cell Line Integrated Molecular Authentication database (CLIMA) version 0.1.201406 (http://bioinformatics.istge.it/clima/clima_search.php)^44^.

### UVB-irradiation of HaCaT cells

For the development of UVB-transformed cell line model, HaCaT cells were seeded (1 × 10^6^ cells/plate) in 60-mm glass Petri-dishes and allowed to grow for 24 h. Thereafter, medium was replaced with phosphate buffer saline (PBS) and cells were exposed to UVB radiation (30 mJ/cm^2^) using UVA/UVB Research Irradiation Unit (Daavlin, Bryan, OH) once a week. Treatment was continued for various time periods viz. 3, 8, 12 and 16 weeks and after each time points, a set of cells treated cells were separated and maintained in regular medium. Derived sublines after attaining stable features were designated as per treatment period viz. 3wk, 8wk, 12wk and 16wk.

### Growth kinetics assay

Parental (NT) and UVB-transformed HaCaT sublines (3wk, 8wk, 12wk and 16wk) were seeded in six-well plates (1 × 10^4^ cells/well). Number of cells was counted using Countess® Automated Cell Counter (Life technology™) on each day up to 8^th^ day to determine the growth rate. Thereafter, growth curve was plotted and cell population doubling time (dt) during exponential growth phase (72–120 h) was calculated using the following formula: dt = 0.693 *t/ln (Nt/N0*), where *t* is time (in h), *Nt* is the cell number at time t, and *N0* is the cell number at initial time.

### Clonogenicity and sphere-formation assays

Anchorage -dependent and -independent clonogenicity assays were performed as described previously^45^,^46^. For sphere-formation assay, single-cell suspensions of HaCaT sublines (1 × 10^3^ cells/well) were seeded in ultra-low attachment 6-well plates (Corning, Inc., Corning, NY) containing sphere-formation medium [DMEM/F12 (1:1), supplemented with B27, bFGF (10 ng/mL) and EGF (10 ng/mL)] and allowed to form spheres for 2 weeks. Following incubation, spheres were counted under Nikon Eclipse microscope (Nikon Instruments Inc. Melville, NY).

### Cell cycle analysis

Cells (5 × 10^5^ cells/well) were synchronized by culturing them in serum-free media as described previously^47^. Subsequently, cells were grown in regular media for 24 h, washed, trypsinized and fixed with 70% ethanol overnight at 4 °C. Post fixation, cells were washed and stained with Propidium Iodide using PI/RNase kit (BD Bio Sciences, San Jose, CA) and analyzed by flow-cytometry on a BD-FACS Canto™ II (BD Bio Sciences). The percentage of cell population in various phases of cell cycle was calculated using Mod Fit LT software (Verity Software House, Topsham, ME).

### Apoptosis assay

Apoptosis assay was performed using PE Annexin V apoptosis detection kit (BD Biosciences) as described earlier^45^. Briefly, control and UVB-transformed HaCaT cells (5 × 10^5^ cells/well) were seeded in 6-well plate. After 24 h, cells were replenished with fresh medium and further allowed to grow for 96 h. Thereafter, cells were harvested, stained with PE Annexin V and 7AAD (7-Amino-Actinomycin-d) solution as per manufacture’s protocol and analyzed by flow cytometry.

### Immunoblot analysis

Total protein from HaCaT and its UVB-irradiated sublines was collected and subjected to immunoblotting as described earlier^45^,^48^. Subsequently, immunodetection was carried out using specific primary antibodies against: Bax, Bcl2, Cyclin D1 (rabbit polyclonal, 1:1000), Slug, Snail, Bcl-xL, E-Cadherin, N-Cadherin (rabbit monoclonal, 1:1000; Cell Signaling Technology, Beverly, MA), Cyclin E, p21 (mouse monoclonal, 1:500), p27 (rabbit polyclonal, 1:500; Santa Cruz Biotechnology, Santa Cruz, CA). Thereafter, blots were incubated with HRP-labeled respective (anti-mouse or anti-rabbit) secondary antibodies (1:2000; Santa Cruz Biotechnology), washed and processed with ECL plus Western Blotting detection kit (Thermo Scientific, Logan, UT). Signal was detected using an LAS-3000 image analyzer (Fuji Photo Film Co., Tokyo, Japan). β-actin was used as loading control (1:20,000; Sigma-Aldrich, St. Louis, MO).

### Motility and invasion assays

Cells (2 × 10^5^ and 5 × 10^4^ for migration and invasion, respectively) were seeded in the top chamber of non-coated (for migration) and Matrigel-coated (for invasion) transwell chamber (BD Biosciences), respectively. Medium supplemented with 10% FBS was added to the lower chamber as a chemoattractant. After 16 h of incubation, non- migrated/invaded cells on the upper surface of the membrane were removed and the migrated/invaded cells on the bottom surface were fixed, stained with Diff-Quick cell staining kit (Dade Behring, Newark, DE), mounted on slide and counted under the microscope in 10 random fields at 200X.

### Phalloidin staining

Phalloidin staining for actin filaments was performed according to previously described procedure^49^,^50^. Briefly, Cells (1 × 10^4^) grown on FluoroDish (World Precision Instruments., Sarasota, FL) for overnight were fixed in 4% formaldehyde in PBS for 10 min at room temperature. Next, cells were washed with PBS and permeabilized in 0.1% Triton X-100 prepared in PBS, for 5 min and blocked with antibody diluent for 45 min. F-actin was selectively labeled with Alexafluor 488 phalloidin (Molecular Probes, Invitrogen, Eugene, OR) for 20 min. After washing cells were mounted using Vectashield mounting medium with DAPI. Immunostaining was observed under Nikon Eclipse TE2000-U fluorescent microscope (Nikon Instruments Inc, Melville, NY).

### Subcutaneous xenograft mouse tumor model and histological analysis

Animal studies were carried out in accordance with the standard principles and procedures approved by the Institutional Animal Care and Use Committee (IACUC) of University of South Alabama. Immunodeficient nude female mice (4- to 6-week old) were purchased from Harlan Laboratories; Prattville, AL. Parental and UVB-transformed (16wk) HaCaT cells (1 × 10^6^ suspended in 50 μL of HBSS medium) were injected into the left flank region of mice (n = 5, each group) to test their tumorigenic potential. Tumor growth was measured using Vernier Caliper once a week up to 21 weeks. At the end point, mice were sacrificed by CO_2_ asphyxiation and autopsied. Tumors recovered from sacrificed mice were weighed and tumor volume was calculated using the formula: π/6 × (smaller diameter)^2^ × (larger diameter). Tissue sections of formalin fixed, paraffin embedded tumors were stained with Hematoxylin and Eosin (H&E) and observed under microscope for histological examination.

### Statistical analysis

All the experiments were performed at least three times independently and data expressed as “mean ± SEM”. Wherever appropriate, the data were also subjected to unpaired two tailed Student’s t-test. p < 0.05 was considered statistically significant.

## Additional Information

**How to cite this article**: Tyagi, N. *et al.* Development and Characterization of a Novel *in vitro* Progression Model for UVB-Induced Skin Carcinogenesis. *Sci. Rep.* **5**, 13894; doi: 10.1038/srep13894 (2015).
